# Remotely Controlled 3D-Engineered Scaffolds for Biomimetic In Vitro Investigations on Brain Cell Cocultures

**DOI:** 10.1002/aisy.202400261

**Published:** 2024-06-03

**Authors:** Daniele De Pasquale, Attilio Marino, Carlotta Pucci, Omar Tricinci, Carlo Filippeschi, Pietro Fiaschi, Edoardo Sinibaldi, Gianni Ciofani

**Affiliations:** Smart Bio-Interfaces, Istituto Italiano di Tecnologia, Viale Rinaldo Piaggio 34, 56025 Pontedera, Italy; Bioinspired Soft Robotics, Istituto Italiano di Tecnologia, Via Morego 30, 16163 Genova, Italy; Department of Neurosurgery, IRCCS Ospedale Policlinico San Martino, Largo Rossana Benzi 10, 16132 Genova, Italy; Department of Neuroscience, Rehabilitation, Ophthalmology, Genetics, Maternal and Child Health (DiNOGMI), University of Genova, Largo Paolo Daneo 3, 16132 Genova, Italy; Bioinspired Soft Robotics, Istituto Italiano di Tecnologia, Via Morego 30, 16163 Genova, Italy; Smart Bio-Interfaces, Istituto Italiano di Tecnologia, Viale Rinaldo Piaggio 34, 56025 Pontedera, Italy

**Keywords:** 3D biomimetic cell cocultures, geometrically engineered scaffolds, in vitro glioma environment, magneto-responsive scaffolds

## Abstract

Most in vitro studies regarding new anticancer treatments are performed on 2D cultures, despite this approach imposes several limitations in recapitulating the real tumor behavior and in predicting the effects of therapy on both cancer and healthy tissues. Herein, advanced in vitro models based on scaffolds that support the 3D growth of glioma cells, further allowing the cocultures with healthy brain cells, are presented. These scaffolds, doped with superparamagnetic iron oxide nanoparticles and obtained through 2-photon polymerization, can be remotely manipulated thanks to an external magnet, thus obtaining biomimetic 3D organization recapitulating the brain cancer microenvironment. From a geometric point of view, the structure is functional to both cell culture on individual unit scaffolds and to tailored cocultures fostered by magnetic-driven unit assembly, also allowing for cell migration thanks to passages/fenestrations on adjacent structures. Leveraging magnetic dragging, for which a mathematical model is introduced, multiple cocultures are achieved, highlighting the high versatility and the user-friendly character of the proposed platform that can help overcome the current challenges in 3D cocultures handling, and open the way to the construction of increasingly biomimetic artificial systems.

## 1 Introduction

Glioblastoma multiforme (GBM) is the most aggressive and deadliest brain tumor, characterized by extreme genotypic and phenotypic variability and high infiltrative capabilities into healthy brain tissues.^[1]^ These features make its treatment extremely difficult, resulting in a very poor prognosis and survival rate.^[2]^ To boost the research advancement in GBM treatment, one of the main goals is to develop tumor models that closely mimic the in vivo tumor features, and that, at the same time, allow for a quick and predictive screening of new therapeutics, reducing the number of in vivo tests needed for further validation.

In vitro models represent a fundamental tool to test the efficacy of new antitumor therapies, as they offer a high throughput and versatile platform to perform a wide set of experiments and allow for a better understanding of the mechanisms of action at the molecular level.^[3]^ Nevertheless, current 2D in vitro models fail to represent the real physiological situation in the tumor tissue in vivo, and they do not take into account the whole tumor microenvironment.^[4]^ These limitations might have strong spillovers when studying complex tumors like GBM, where the microenvironment has a huge impact on tumor progression and drug efficacy, and the crosstalk between cancer and healthy cells cannot be overlooked.^[5]^ Tumor masses grow in three dimensions: drug diffusion observed in 2D cultures may thus significantly differ in 3D solid tumors;^[6]^ moreover, 2D models do not mimic the cell–cell interactions and cell signaling pathways occurring in 3D. Eventually, the multicellular layers of the 3D tumor decrease the gas exchanges and the nutrient supply, thus compromising the penetration of drugs and, therefore, their effectiveness.^[7,8]^ Hypoxia due to a decreased gas exchange is a phenomenon that occurs when solid tumors have a diameter larger than 200 μm; in these conditions, tumor cells start to express proangiogenic factors, among which the hypoxia-inducible factor (HIF1α), with the aim of stimulating blood vessels growth.^[7,9]^ Consequently, solid tumor cells change their gene expression and phenotype: studies in rodents demonstrated that O_2_ depletion, associated with nutrient deprivation, promotes tumor malignancy.^[10–12]^ Recent works moreover demonstrated that the inner niche of solid tumors contains tumor stem cells, which are the main cause of drug resistance and recurrence.^[13]^ For all of these reasons, drug research through more realistic 3D tumor models is extremely important to better simulate the tumor features and to improve anticancer treatment protocols

As mentioned, another important aspect to consider when studying GBM is the interaction of cancer cells with the microenvironment, composed of the healthy cells that populate the brain.^[14]^ It has been previously reported that their interactions affect drug efficacy and tumor progression,^[14]^ and thus efficient in vitro models should take into account also this important interplay, by coculturing tumor cells with representative healthy cells, such as astrocytes, pericytes, neurons, microglia, or brain endothelial cells.

Several studies concerning tumor in vitro models lack the tumor–stroma interactions; the combination of coculture systems with 3D tumor spheroids offers instead a better representation of cancer complex biology and a powerful tool to better predict not only the efficacy of an antitumor treatment, but also its possible side effects on healthy cells, an aspect that is often poorly investigated in cancer research.^[15]^ Finding a strategy for accurate manipulation of cells grown in 3D conditions would bring substantial advantages for biomimetic in vitro coculture systems suitable for GBM investigations. Several types of magnetically driven, scaffold-free 3D spheroids have been already proposed, and they all rely on the administration of ferrous nanoparticles to the cells or the spheroids.^[16–21]^ However, this approach might induce alterations to the cellular physiology, due to the interaction of the nanoparticles with the cells. Moreover, biodegradable scaffolds including magnetic nanoparticles were also proposed, e.g., for treating bone carcinoma via hyperthermia:^[22]^ conversely, our study addresses the development of nonbiodegradable scaffolds, so that magnetic nanoparticles cannot be released in the surrounding environment. Moreover, the use of two-photon polymerization (2-PP) allows for a general greater fabrication accuracy.^[23]^ Barin et al. proposed a 3D scaffold anchored to the culture surface to study glioma cellular features in terms of nucleus shape, arrangement of tubulin fibers, and expression of epidermal growth factor (EGF) receptors with respect to traditional 2D cultures.^[24]^ Akolawala et al. by mimicking the geometry of brain blood vessels, designed a 3D-engineered scaffold that supported the growth of the GBM cell line U-251 to study the effects of proton beam therapy.^[25]^ Nevertheless, in that study, the interaction with other nontumor cells usually composing the tumor niche was not taken into account, and the scaffolds could not be remotely controlled.

In previous works from our lab, moreover, we showed that magnetic microstructures obtained both with doping with superparamagnetic iron oxide nanoparticles (SPIONs)^[26]^ or with a postfabrication coating with magnetic materials,^[27]^ represent a very efficient tool for the investigation of the brain tumor microenvironment. Here, we extend this approach to achieve “direct” cocultures of different cell lines (i.e., cultures where more cell populations come physically into contact with each other), previously grown on individual magneto-responsive scaffolds, which are then combined through magnetic guidance. We demonstrated the versatility of this technological approach, allowing the simple and reproducible production of scaffolds with desired geometries, as well as their handling. In particular, two different types of scaffolds were designed and fabricated by 2-PP, named “cage-like” scaffolds and “net-like” scaffolds, according to the adopted geometry (expressly designed to optimize cellular interactions), doped with SPIONs and thus magnetically responsive. We investigated their biocompatibility and their ability to be driven by a remote magnetic field, following seeding with commercial U87-MG glioblastoma line, primary patient-derived GBM cells, primary human astrocytes, primary human microglia, and primary human pericytes. A theoretical model was moreover developed to quantitatively characterize the response of the magneto-responsive structures. Finally, cocultures were successfully achieved following microscaffolds assembly. The proposed scaffolds could serve as standardized tools for the development of in vitro models for cell cocultures.

## 2 Experimental Section

### 2.1 Scaffold Fabrication

**Figure 1:**
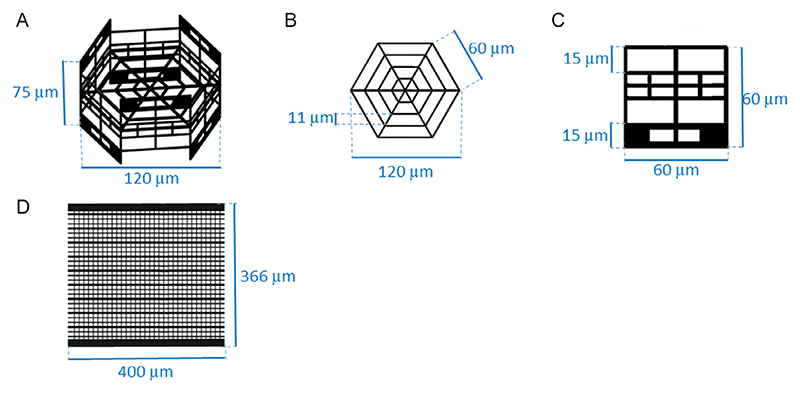
Schematic drawings of the structures. A) Overall 3D scheme of the MR-CS and relative sizes. B) Representation of the internal grid of the MR-CS and relative sizes. C) Representation of one of the lateral sides of the MR-CS and relative sizes. D) Scheme of the MR-NS with relative sizes (top view).

Magnetically-responsive cage-like scaffolds (MR-CSs) and magnetically-responsive net-like scaffolds (MR-NSs) had been designed not only to stably host cells during the seeding, but also to allow for their growth and migration between neighboring scaffolds, and to facilitate cocultures with the help of an external magnet. The structures were sized (Figure 1A–C) according to a quantitative analysis of cell diameter in suspension (i.e., the situation occurring during the seeding), provided in Table S1, Supporting Information. Considering the different cell diameters, the fenestrations and the grids of the scaffolds were sized and positioned in such a way that cells can perform active migration, by overcoming the relatively narrower fenestrations aligned with the seeding plane, which in turn prevent passive escape of the cells. MR-CSs had the shape of a hexagonal prism, mirrored along the *Z* axis, with six 75 μm-high perimeter cylindrical pillars of 2 μm diameter, and with an inner grid of 120 μm diagonal length, consisting of three concentric hexagons separated by 11 μm slits (narrower than the diameter of the seeded cells). Each sidewall of the hexagon had a width of 60 mm, with fenestrations of 27 μm × 15 μm large enough to allow migration of cells from the cage, and smaller fenestrations of 7.3 μm × 12 μm to temporarily block cells during seeding.

The structure of the MR-NSs was based on a rectangular grid of 366 μm × 400 μm (Figure 1D), supported by 24 cubic supports (2 μm edge length), regularly spaced underneath the perimeter of the considered rectangular scaffolds. The latter presented two end-sides of 400 × 20 × 4 μm to increase its ferromagnetic mass, thus favoring responsiveness to magnetic fields, and lines inside the grid of 400 μm × 6 μm × 6 μm alternating with lines of 400 μm × 3 μm × 6 μm. Perpendicularly, lines of 358 μm × 2 μm × 2 μm completed the formation of the network.

To emphasize the versatility of the proposed technique, a microscale “space shuttle” structure had been designed and produced with the same approach; details are reported in Supporting Information (Figure S1).

All the aforementioned scaffolds were designed with the software AutoCAD, while the 3D printing parameters for the scaffold fabrication were set on the nanowrite software (Nanoscribe GmbH).

The nontoxic negative photoresist IP-L 780 (Nanoscribe GmbH) was doped with 10 μg mL^−1^ of SPIONs of 3 nm in diameter (US Research Nanomaterials, Inc.), the biocompatibility of which had been already tested in previous works.^[28,29]^ The mixture was sonicated for 5 min in an ice bath with a Bandelin ultrasonic probe (8 W) to obtain a homogeneous IP-L 780/SPIONs dispersion.

The fabrication of the magneto-responsive structures was carried out by means of Photonic Professional GT2 system (Nanoscribe GmbH) equipped with a femtosecond laser system with a center wavelength of 780 nm (Toptica laser source). Structures were printed from a drop of IP-L 780/SPIONs dispersion, cast on a glass coverslip of 3 cm in diameter (VWR), and exposed to the laser beam through an oil objective (63×, NA 1.4), using a writing speed of 40 mm s^−1^ and a laser power of 40 mW. The corresponding light intensity (*I*) at the focal plane is 1.37 × 10^13^ W cm^−2^. This value could be derived considering the Gaussian distribution of the light intensity that can be expressed as $I=\frac{2PT}{Rw^{2}\pi \tau }$,^[30]^ where *P* is the average power (40 mW), *T* is the objective transmittance (100%), *R* is the pulse repetition rate (80 MHz), *τ* is the pulse duration (85 fs). The value of *w* (165 nm), that is the radius at the beam waist, can be obtained from $w=\frac{2f\lambda M^{2}}{\text{π}\phi }$,^[31]^ where *f* is the focal distance (360 μm), *λ* is the laser wavelength (780 nm), *M*^2^ is the beam quality factor (1.2), *ϕ* is the beam diameter at the lens (1.3 mm).

After the 2-PP process, the structures were developed in dark conditions at room temperature for 30 min with the SU-8 Developer (Microchemicals GmbH), and washed by immersion for 15 min with isopropyl alcohol; at the end of manufacturing procedure, the scaffolds were still anchored to the glass were stored in ultrapure water until use.

For scanning electron microscope (SEM) imaging, the scaffolds were gold-sputtered at 30 mA for 60 s by a Quorum Tech Q150RES Gold Sputter Coater, and thereafter imaged with a Helios NanoLab 600i FIB/SEM (FEI).

### 2.2 Model-Based Estimate of the Magneto-responsive Volume Fraction

To investigate the magnetic response of the doped photoresist, microspheres characterized by three different radius sizes (6.4 ± 0.4, 13.3 ± 2.0, and 29.2 ± 1.3 μm) were designed (using AutoCAD), by adding small cylindrical supports (1 μm in diameter) for their anchoring on the substrate (Figure S2, Supporting Information). The spheres were fabricated via 2-photon polymerization, by using the same photoresist and fabrication parameters adopted for the MR-CSs and MR-NSs. After the development step, the printed spheres were collected with a pipette and placed into a 10 cm-diameter Petri dish filled with ultrapure water for magnetic dragging analyses under an inverted microscope (Eclipse Ti-E, Nikon, 4× objective). A parallelepiped magnet (40 × 20 × 10 mm^3^, NdFeB 1.30 T remanence, Impressum WebCraft GmbH) was placed at 12.5 mm from the sphere and its attraction movement was recorded with the microscope camera in bright field modality. Considering the relative importance between inertial and viscous effects at the relevant length scale (namely the sphere characteristic size), magnetic dragging induces a constant speed motion for the sphere over the observed motion range: we computed the sphere speeds based on the recorded flight time to span 212 μm from the resting point. The as-obtained experimental data were then used for characterizing the magnetic response of the doped photoresist, and in particular its magneto-responsive volume fraction, by means of a simple mathematical model, as described hereafter.

The magnetic field generated by the adopted 10 × 40 × 20 mm^3^ brick magnet was modeled by a multidipole approximation,^[32]^ by assuming a three-dimensional array of elementary bricks with volume *V*_b_, thus dipole moment ${\vec{m}}_{\text{b}}=\vec{M}\cdot V_{\text{b}}$, with magnetization intensity *M* directly derived from magnet remanence (1.30 T). The number of elementary bricks was increased up to obtaining discretization-independent results (Figure S3, Supporting Information), in particular in terms of relative variations of magnetic induction components below 0.1% at the initial sphere position (the sphere was initially placed at a distance of 12.5 mm from the magnet north-pole face; see Figures S4, Supporting Information, for a schematic). The derived magnetic field intensity at the initial sphere position O, namely *H*|_O_ ≅ 7.55 × 10^4^ A m^−1^, permitted to rule out SPIONs saturation (occurring at 3.10 × 10^5^ A m^−1^, based on the saturation magnetization per unit mass reported in Tapeinos et al.^[33]^ and assuming a SPIONs density of 5 g cm^−3^, as commonly for iron oxide). Indeed, in light of the limited dragging span (212 μm), magnetic field variations could be considered as negligible (for instance, the relative variation of *H*^2^, also relevant for subsequent derivations, was below 3% over the whole traveled span).

Each SPION was treated as a point-dipole, induced by the local value of the external magnetic field created by the brick magnet. Below the saturation limit, the adopted point-dipole model, which was previously applied for both theoretical^[34]^ and applicative studies also involving experimental validation,^[35]^ leads to the following expression for the magnetic force ${\vec{f}}_{\text{m}}$ acting on a sphere: ${\vec{f}}_{\text{m}}=\beta_{\text{m}}V_{\text{s}}\mu \varphi \vec{H}\cdot \text{grad}(\vec{H})$, where *β*_m_ denotes the magneto-responsive volume fraction, $V_{\text{s}}=(4/3)\pi r_{\text{s}}^{3}$ is the sphere volume (*r*_s_ denoting the radius), *μ* represents the magnetic permeability of the resist surrounding the magnetic nanoparticles, and $\vec{H}$ denotes the external field. Moreover, *φ* = 3(*χ*_NP_ – *χ*) /[(*χ*_NP_ – *χ*) + 3(*χ* + 1)], where *χ* = (*μ/μ*_0_) − 1 and *χ*_NP_ denote the magnetic susceptibility of resist and magnetic nanoparticles, respectively (with *μ*_0_ = 4*π* × 10^−7^ T m A^−1^ denoting vacuum permeability). Furthermore, given the well-known vector identity $\text{grad}(\vec{a}\cdot \vec{b})=\vec{a}\cdot \text{grad}\vec{b}+\vec{b}\cdot \text{grad}\vec{a}+\vec{a}\wedge \text{curl}\vec{b}+\vec{b}\wedge \text{curl}\vec{a}$, taking $\vec{a}=\vec{b}=\vec{H}$ with $\vec{H}$ curl-free, the magnetic force can be recast as follows: ${\vec{f}}_{\text{m}}=(2/3)\pi \beta_{\text{m}}r_{\text{s}}^{3}\mu \varphi \text{grad}\left(H^{2}\right)$. In addition, by considering the sphere motion along the *x*-direction (see the schematic in Figure S4, Supporting Information), consistently with the carried out experiments, and by recalling the aforementioned negligible variation of the magnetic field (and gradient) over the dragging span, the *x*-component of the magnetic force becomes ${f_{\text{m}x}≅(2/3)\pi \beta_{\text{m}}r_{\text{s}}^{3}\mu \varphi \left(\partial H^{2}/\partial x\right)|}_{\text{O}}$ (where subscript O denotes the initial sphere position, as above).

Finally, given the low Reynolds number for the problem at hand, we assumed that magnetic pulling was directly balanced by the classical Stokes drag force acting on the sphere, i.e., ${\vec{f}}_{\text{m}}+{\vec{f}}_{\text{d}}=\vec{0}$, with ${\vec{f}}_{\text{d}}=-6\pi \eta_{\text{f}}\;r_{\text{s}}\vec{u}$, where *η*_f_ represents liquid viscosity and $\vec{u}$ denotes sphere velocity. Along the *x*-direction, the Stokes drag force thus reads *f*_d*x*_ = −6*πη*_f_ *r*_s_ *u*, where symbols are understood, and the related momentum balance *f*_m*x*_ *+ f*_d*x*_ = 0 straightforwardly provides the following scaling law for the sphere speed *u* versus the sphere radius *r*_s_

(1) $$u≅C_{2}\;r_{\text{s}}^{2}$$

with

(2) $$C_{2}={\frac{\beta_{\text{m}}}{9}\frac{\mu }{\eta_{\text{f}}}\left[\frac{3\left(\chi_{\text{NP}}-\chi \right)}{\left(\chi_{\text{NP}}-\chi \right)+3(\chi +1)}\right]\frac{\partial H^{2}}{\partial x}|}_{\text{O}}$$

The aforementioned model was implemented in MATLAB (The Mathworks). To this purpose, considering that the adopted resist is basically a mixture of acrylic polymers and a photoinitiator, we assumed *χ* ≅ 0 (whence *μ* ≅ *μ*_0_), thus making the (nondimensional) value of the square-bracketed term in Equation (2) approach 3, given that *χ*_NP_ >> 1 for SPIONs (being, e.g., order of 10^5^ for iron). Furthermore, in light of the adopted water-like fluid, we assumed *η*_f_ ≅ 10^−3^ Pa s, and we numerically computed the gradient by using centered (2nd-order accurate) finite differences,^[36]^ still based on a grid refined enough to obtain discretization-independent results. Finally, computational cost (on a common desktop) was negligible (order of a minute), thanks to the explicit analytical form of the involved expressions.

### 2.3 Cell Seeding in the Scaffolds

U87-MG (ATCC HTB-14) and GFP-expressing U87-MG cells (Cellomix SC-1495) were cultured in T75 flask with high-glucose Dulbecco’s modified Eagle’s medium (DMEM, Sigma-Aldrich) supplemented with 10% heat-inactivated fetal bovine serum (FBS, Sigma-Aldrich) and 1% penicillin-streptomycin (100 IU mL^−1^ of penicillin and 100 μg mL^−1^ of streptomycin, Gibco); this preparation will be defined as “complete medium.” After a standard trypsinization procedure, 4 × 10^4^ cells in 50 μL of medium were dropped on the MR-CSs array, still attached on a glass surface, in a Petri dish (3 cm of diameter); after 2 h, the Petri was filled with 2 mL of complete medium. Cell proliferation within the MR-CSs after 4 days of incubation at 37 °C allowed the development of remotely controllable spheroids of ≈200 μm in diameter. Spheroids obtained with this approach (that from now in advance we will indicate as “method 1”) were mechanically removed with a 1000 μL pipette tip and collected through a brief aspiration. They were then transferred to multiwell plates, with 300 μL of complete medium, for further experiments.

The “method 2” envisioned instead the seeding of cells (about 100 cells were in 50 μL) in 2% agarose gel-coated 96-well plates containing one MR-CS per well. After 4 days of proliferation, magnetically controllable spheroids of ≈200 μm were obtained, collected with a 1000 μL pipette, and transferred to multiwell plate for experiments.

Eventually, 3D control cultures in absence of microscaffolds were obtained by seeding cells on agarose-coated plates.

Cells were labeled with TRITC-phalloidin to stain the F-actin microfilaments of the cytoskeleton, thus allowing for their detection through fluorescence microscopy. After fixation with 4% paraformaldehyde (PFA, Sigma-Aldrich) for 20 min at 4 °C, the samples were washed three times with DPBS (Gibco) and permeabilized with Triton-X 100 1:1000 solution (Sigma-Aldrich) for 30 min, before treatment with 2 mL of TRITC-phalloidin 1:200 (Sigma-Aldrich) and Hoechst 33 342 1:1000 (Invitrogen, for nucleus counterstaining) in DPBS and for 1 h at 37 °C. Images of the samples were acquired with a laser scanning microscope (C2s, Nikon). An analogous procedure has been followed for the staining of the other cultures described in this work.

Patient-derived GBM cell cultures have been obtained in our laboratory in agreement with procedures approved by the local ethical committee (Registro CER Liguria 341/2019) by isolating cells from surgical resections of GBM from San Martino hospital (Genova, Italy); briefly, the resections were washed with DPBS without Ca^2+^ and Mg^2+^ (Gibco) and dissected with a scalpel into small pieces of about 1 mm^3^, incubated with 0.05% trypsin-EDTA for 10 min, and sieved with 70 μm-sieves (Corning). The filtered cell suspension was later placed in culture in flasks previously coated with 5 mL of synthetic extracellular matrix (Geltrex 5%, Gibco) at 37 °C with serum-free stem cell media that preserves the stemness of primary GBM cells,^[37,38]^ and composed of 48% DMEM F12 (Sigma-Aldrich), 47% DMEM high-glucose (Sigma-Aldrich), 2% B-27 nutrient supplement (Thermo Fisher), 1% penicillin-streptomycin (100 IU mL^−1^ of penicillin and 100 μg mL^−1^ of streptomycin, Gibco), 1% insulin solution from bovine pancreas (10 mg mL^−1^, Sigma-Aldrich), 1% L-glutamine (Gibco), 10 ng mL^−1^ fibroblast growth factor (bFGF Sigma-Aldrich), 20 ng mL^−1^ EGF (Sigma-Aldrich), and 10 ng mL^−1^ heparin sodium salt from porcine intestinal mucosa (Sigma-Aldrich).

For seeding, method 1 was followed; primary GBM cells (3 × 10^4^ cells in 50 μL of medium promoting the maintenance of stem conditions) were dropped on the MR-CS array for spheroids development. The control spheroids were obtained through spontaneous generation of spherical structures by GBM primary cells maintaining the culture for 30 days in stemness conditions. Spheroids with 200–300 μm of diameter were collected and fixed with 4% PFA as previously reported.

Primary human astrocytes (Alphabioregen HBMP-202) or GFP-expressing primary human astrocytes (Alphabioregen HBMP201-F) were seeded in MR-NSs. The cultures were maintained in Astrocytes Growth Medium (Alphabioregen) and, for seeding into MR-NSs, 3 × 10^4^ cells in 100 μL of medium were placed in a Petri dish with an array of 8 scaffolds; 2 h after inoculation, further 2 mL of medium was added and the cells allowed to growth in the MR-NSs for 4 days before any further use. After the fixation procedure (PFA 4%), the samples were stained with TRITC-phalloidin and Hoechst 33 342, as previously reported.

Primary human brain microglia (HBPM, Alphabioregen PHM001) were cultured in Petri dish with Alpha-Glia Expansion Medium (Alphabioregen) and 1% penicillin-streptomycin (100 IU mL^−1^ of penicillin and 100 μg mL^−1^ of streptomycin, Gibco); following standard trypsinization, 100 μL of medium containing 3 × 10^4^ cells were dropped in a 3 cm-diameter Petri dish containing 8 MR-NSs organized as an array and allowed to growth for 3 days before any further experiment. After the fixation procedure (PFA 4%), the samples were stained with TRITC-phalloidin and Hoechst 33 342, as previously reported.

Primary human brain microvascular pericytes (HBVP ScienCell, 1200-SC) were cultured in 10 cm Petri dish with Pericytes Medium (Pericytes Basal Medium + Pericytes Growth Supplement, Sciencell). Following standard trypsinization, 100 μL of medium containing 3 × 10^4^ cells were dropped in a 3 cm-diameter Petri dish containing a MR-NS array of 8 scaffolds, and allowed to grow for 2 days before any further experiment. After the fixation procedure (PFA 4%), the samples were stained with TRITC-phalloidin and Hoechst 33342, as previously reported.

### 2.4 Cell Viability Investigation

Patient-derived GBM cells were seeded into the MR-CSs as previously reported; in parallel, control spheroids (without scaffolds) were prepared by culturing cells on agarose-coated surfaces until the obtainment of spheroids of about 200 μm in diameter. After 4 days of cultures, 5 spheroids grown in suspension and 5 spheroids developed in the MR-CSs were collected and transferred into a 48-well plate (one spheroid per well), and the metabolic activity was assessed by incubating the samples in 300 μL of a 1:20 solution of WST-1 (Roche) in complete medium without phenol red (Gibco). After 60 min, the supernatants were collected and their absorbance was read at 560 nm with a Victor X3 multiplate reader. The same operations were performed to test the metabolic activity after 72 h of spheroid development. Data have been analyzed by subtracting the blank (WST-1 solution) from the measurements and setting to 100% the values obtained for the control cultures.

The culture of primary human astrocytes in MR-NSs was performed following the previously reported protocol. To have a control culture of primary human astrocytes (2D), 70 cells were seeded in 96-well plates. After 4 days, WST-1 test was carried out as previously described, and repeated after further 72 h.

The cell viability was also tested by Live and Dead assay (LIVE/DEAD Cell Imaging Kit, Thermo Fisher); the control spheroids and spheroids grown within MR-CSs were collected and stained with calcein-AM 1:4000 (to highlight live cells in green), ethidium homodimer-1 1:1000 (to highlight dead cells in red), and Hoechst 33 342 1:1000 (for nuclear staining, in blues) in complete medium and incubated for 40 min at 37 °C. After washing in PBS, spheroids were maintained in a complete medium without phenol red, and images were acquired with confocal laser scanning microscope (C2s, Nikon). The count of ethidium homodimer-1 positive cells compared to the number of total cells labeled with Hoechst was carried out using ImageJ software.

The effects of magnetic manipulation were investigated considering 6 spheroids of U87-MG developed in MR-CSs and human astrocytes cultured in 6 MR-NSs; the presence of the external magnet, exploited for the scaffolds manipulation, was considered as well (6-h exposure). Three samples were collected and placed in a well (contained in a 24-well plate), and incubated with 400 μL of WST-1 solution 1:11 in complete phenol red-free medium, for 30 min in the case of MR-CS spheroids, and for 50 min for MR-NSs with human astrocytes; the WST-1 test was then carried out as previously described.

### 2.5 Cell Markers Expression Investigation

Two markers have been investigated: Ki-67, indicative of an active proliferative status,^[39]^ and SOX2, a marker of stemness.^[40]^ 5 GFP-expressing U87-MG spheroids obtained in MR-CSs and 5 conventional control spheroids have been fixed with 4% PFA (20 min at 4 °C), permeabilized with a Triton-X 100 solution (1:1000) for 1 h, and incubated for 30 min with goat serum 10% in DPBS (GS, Sigma-Aldrich). 1 mL of 1:200 solution of primary rabbit antibody against Ki-67 was used for a 2 h incubation at 37 °C. After three washing steps with 10% GS, an antirabbit TRITC-conjugated secondary antibody (1:200, Invitrogen) was exploited for incubation at 37 °C for 90 min. Nuclei were counter-stained with Hoechst 33342 1:1000 in DPBS for 20 min. Images were acquired with a confocal laser scanning microscope (C2s, Nikon).

An analogous procedure was followed for 4 samples obtained from patient-derived cells in MR-CSs and conventional spheroids cultured in stemness conditions. In this case, immunofluorescence for SOX2 detection has been performed by using a primary rabbit anti-SOX2 antibody (1:200, GeneTex) and a secondary FITC-conjugated goat antirabbit antibody (1:300, Invitrogen) in 10% GS solutions.

Semiquantitative analyses were performed by counting the number of Ki-67^+^ cells compared to total labeled cell nuclei and counting the number of SOX2^+^ nuclei compared to total Hoechst-labeled nuclei using ImageJ software.

### 2.6 Migratory Capability

Control spheroids (*n* = 3) of ≈200 μm of diameter and 3 spheroids within MR-CSs were obtained with GFP-expressing U87-MG using the “method 2”; then, they were collected, stained with Hoechst 1:1000, and placed individually in 24-well plates (Ibidi) in 400 μL of complete medium without phenol red for microscope acquisitions. Cells migrating out of the structures were analyzed after 6 and 24 h, by assessing the surface area covered by the cells by using ImageJ.

To evaluate the cell invasion capacity in simple coculture conditions, 3 replicates of GFP-expressing U87-MG spheroids (either control or cultured in MR-CSs) were collected, the nuclei were stained with Hoechst to identify single cells during migration, and placed on a 2D culture of SH-SY5Y differentiated cells (ATCC CRL-2266). Concerning the latter, 1.5 × 10^4^ cell cm^−2^ were seeded in 24-well plate (Ibidi) in 500 μL of DMEM F12 (Sigma-Aldrich) supplemented with 10% FBS (Sigma-Aldrich), 1% glutamine (Gibco), and 1% penicillin-streptomycin (Gibco); after 24 h, differentiation was induced by replacing the medium with high-glucose DMEM (Sigma-Aldrich), 1% FBS (Sigma-Aldrich), 1% penicillin-streptomycin (Gibco), and 10 μM retinoic acid (Thermo Scientific). The neuron-like SH-SY5Y-derived cells were stained with DiI 1:2000 (Invitrogen) and Hoechst 1:1000 for 40 min at 37 °C in complete medium; thereafter, after a washing step with DPBS, the wells were filled with DMEM 1% FBS without phenol red, and control spheroids or MR-CSs were added. The confocal acquisitions were carried out at 0, 6, and 24 h, to analyze with ImageJ cells migrating from the spheroids.

### 2.7 Remote Control of Scaffolds and Assembly of Cocultures

#### 2.7.1 Remote Control Experiments

The magneto-responsiveness of the scaffolds was tested on both “empty” and cell-populated MR-CSs; a parallelepiped magnet (40 × 20 × 10 mm^3^, NdFeB 1.30 T, Impressum WebCraft GmbH) was placed and moved at ≈20 mm from the magneto-responsive scaffolds to drive the structures. MR-CSs were placed in a 3 cm diameter Petri dish filled with 1 mL of DPBS. Time-lapse videos were acquired with the aid of a fluorescence microscope by exploiting the autofluorescence of the IPL-780 photoresist, during the manipulation with the remote magnet. When cells (U87-MG) were present on the scaffolds, tracking was allowed owning to the F-actin and nuclei staining. Similar experiments have been performed with the “shuttle-shaped” scaffolds, populated with GFP-expressing U87-MG cells (Figure S1, Supporting Information).

Cell viability was also confirmed upon exposure to the magnetic field; 6 MR-CS spheroids with U87-MG and 6 MR-NSs with human astrocytes were obtained as reported before and collected in two different 30 mm diameter petri dish (Corning), and WST-1 assay performed as previously described after a 6 h exposure to the permanent magnet.

#### 2.7.2 Cocultures Examples

Cocultures of GBM spheroids + human neurons have been obtained with GFP-expressing U87-MG in MR-CSs and differentiated human SH-SY5Y. The two cultures were put in contact and, after 24 h, the samples were fixed and immune-stained for β3-tubulin detection by using a primary rabbit anti-β3 tubulin antibody (1:200, Abcam) and a TRITC antirabbit secondary antibody (1:200, Invitrogen). Nuclei were counterstained with Hoechst before confocal acquisition.

Cocultures of U87-MG spheroids + human astrocytes + human neurons were obtained by using GFP-expressing U87-MG spheroids developed in MR-CSs and GFP-expressing primary human astrocytes grown in MR-NSs, obtained as reported before. The two typologies of microstructures were mechanically detached with a pipette and transferred to a Petri dish containing a culture of differentiated SH-SY5Y cells. With the help of an external magnet, an assembly of the three cultures was obtained. Cultures were eventually fixed and immune-stained for β3-tubulin detection.

Cocultures of U87-MG spheroids + human microglia + endothelial cells were obtained. In this case, human brain endothelial cells hCMEC/D3 (Millipore) were cultured in EndoGRO-VEGF (Millipore) medium with 1% penicillin-streptomycin. 1.5 × 10^4^ cells cm^−2^ were seeded on a 3 cm-diameter coverslip and, after 24 h, MR-NSs seeded with microglia and MR-CSs bearing glioma cells were magnetically put in contact with the endothelial culture. Staining of F-actin and nuclei was performed before confocal imaging.

### 2.8 Statistical Analysis

All statistical comparisons were performed with the Excel software (Microsoft) by *t*-test; differences were considered significant when *p* < 0.05.

## 3 Results and Discussions

### 3.1 Magneto-Responsive Scaffolds

**Figure 2:**
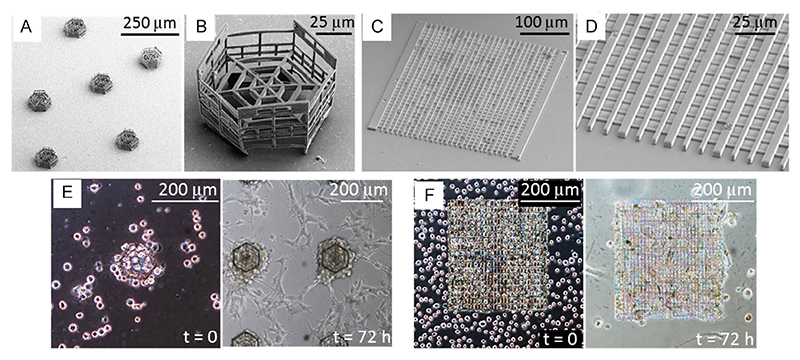
Representative SEM and optical images of the structures developed in this work. A) SEM acquisition of an array of MR-CSs. B) SEM acquisition of a single MR-CS. C) SEM acquisition of an MR-NS. D) Zoomed detail of the MR-NS. E) Optical microscope acquisition of U87-MG cells seeded in the MR-CSs and their relative growth after 72 h. F) Optical microscope acquisition of human astrocytes seeded in the MR-NSs and their growth after 72 h.

Figure 2 depicts an overview of the structures obtained in this work: the provided SEM imaging confirms the successful and reproducible printing even in the presence of nanoparticle-doped resist.

The design of the MR-CSs was chosen to host GBM cells and allow their maintenance inside the scaffold during the seeding phase, support their growth in 3D and, at the same time, allow the migration of the cells through the fenestrations from the scaffold toward the external environment (Figure 2A,B). Similarly to MR-CS, the structure of MR-NSs (Figure 2C,D) has been also designed to avoid cell dispersion during seeding but, at the same time, to allow cell migration from the scaffolds, ideally providing support from healthy cells to the growth and maintenance of glioma.^[41,42]^

Figure 2E shows how the MR-CSs are able to host U87-MG cells inside the cages during the seeding operation, while Figure 2F depicts how the MR-NSs allow the anchoring of the human astrocytes during the first seeding stage. Both images show that after 72 h, the cells efficiently grow on both types of scaffolds.

### 3.2 Model-Based Estimate of the Magneto-Responsive Volume Fraction

**Figure 3:**
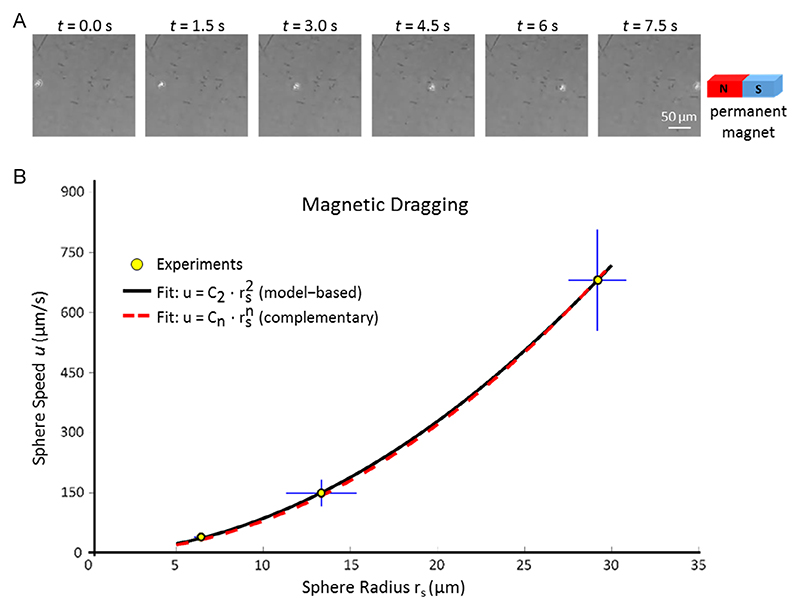
Dragging speed versus radius of spherical samples, used to estimate the magneto-responsive volume fraction of the doped resist used for scaffold fabrication. A) Representative time-lapse images of the movement of the spheres responding to the magnetic field of an external magnet. B) Dragging of magneto-responsive spheres obtained by two-photon lithography: speed *u* versus sphere radius *r*_s_. Experimental data are denoted by circles: three sizes are shown, corresponding to a radius *r*_s_ ≅ 6.4, 13.3, and 29.2 μm. The model-based trend $\left(u\propto r_{\text{s}}^{2}\right)$, represented by the solid curve, accurately fits the data (*R*^2^ = 0.9996). The complementary fitting trend $\left(u\propto r_{s}^{n}\right)$ supports the pursued modeling approach (*n* ≅ 1.93, *R*^2^ = 0.9999).

The dragging experiments with the magneto-responsive spheres (Figure 3A) showed that structures with larger sizes reach higher speeds (680.8 ± 126.9 μm s^−1^ for 29.2 μm diameter spheres, 165.2 ± 29.9 μm s^−1^ for 13.3 μm diameter spheres, and 39.5 ± 7.9 μm s^−1^ for 6.4 μm diameter spheres). Based on such experimentally recorded speeds, the aforementioned mathematical model permitted to estimate the magneto-responsive volume fraction of the doped photoresist used for two-photon lithography, as detailed in the sequel. First, Equation (1) was used to fit the experimental data, resulting in accurate (*R*^2^ = 0.9996) fitting. To further assess the suitability of the introduced model, we also performed a complementary fitting, by assuming the more general trend $u≅C_{n}\;{\text{r}}_{\text{s}}^{n}$: the derived parameter value *n* ≅ 1.93 (with *R*^2^ = 0.9999) supported model derivation (Figure 3B). The parameter value *C*_2_ ≅ 0.80 μm^−1^ s^−1^ obtained by fitting the data with Equation (1) was then used for estimating *β*_m_ through Equation (2), thus obtaining *β*_m_ ≈ 1.9 × 10^−3^. In spite of the simplifications introduced for model derivation, this result suggests that SPIONs well disperse in the nanocomposite. Indeed, considering, for the sake of illustration, a cubic lattice with edge length 𝓁 and having spherical SPIONs of radius *r*_s_ at its vertices, one would get *𝓁/r*_s_ = (4*π*/3*β*_m_)^1/3^ ≈ 13. This result, combined with the experimental findings reported in this study, suggests the possibility of effectively manipulating magneto-responsive structures by 2-PP for cell coculture applications.

### 3.3 Cell Seeding and Compatibility Testing

**Figure 4:**
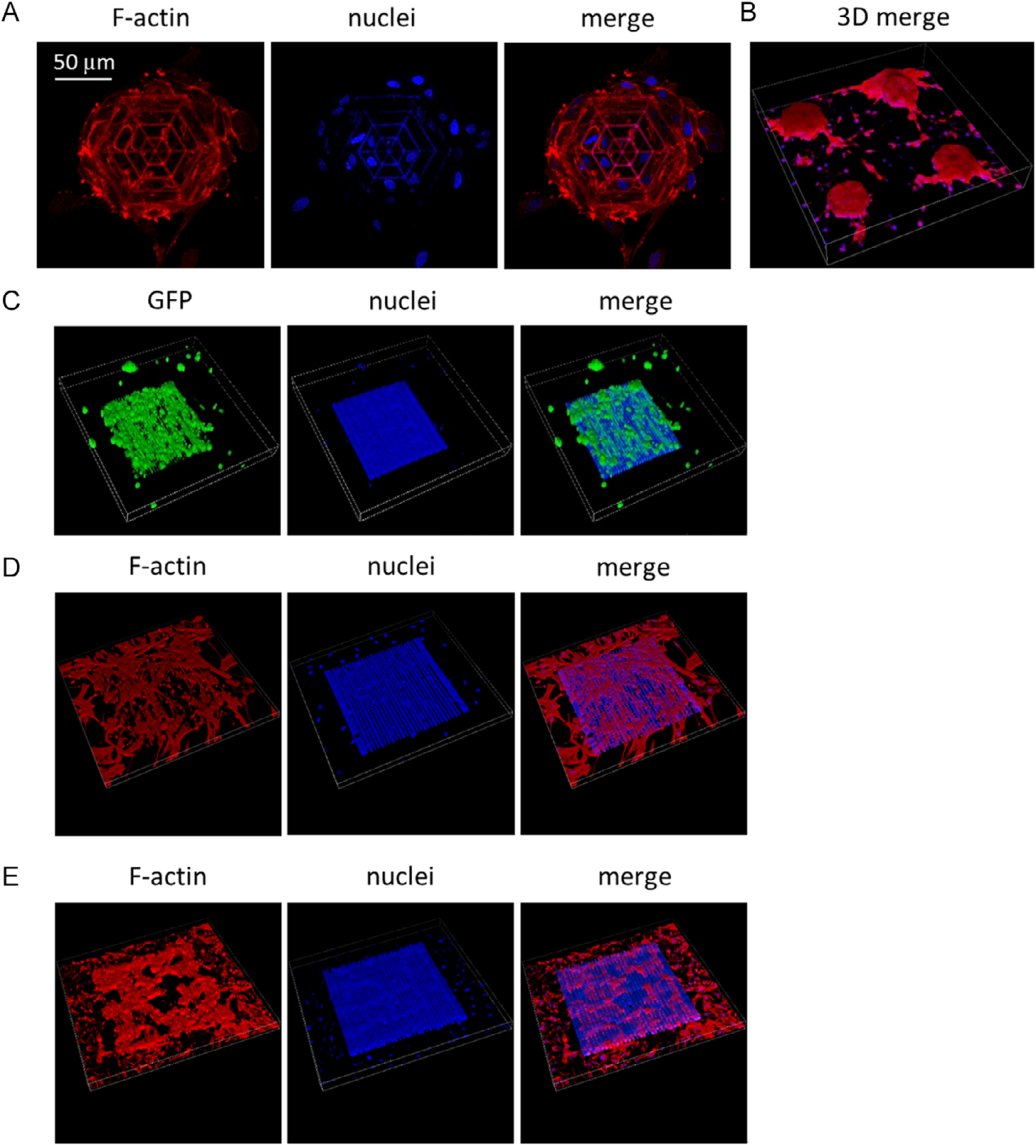
Cell cultures in magneto-responsive scaffolds. A) Medial plane image of a U87-MG spheroid in MR-CS; B) 3D acquisition volume of *x* = 635.8 μm, *y* = 635.8 μm, *z* = 102.6 μm of the spheroids obtained within MR-CSs; C) 3D acquisition volume of *x* = 702.4 μm, *y* = 702.4 μm, *z* = 73.6 μm of GFP-expressing primary human astrocytes in MR-NSs; D) 3D acquisition volume of *x* = 576.8 μm, *y* = 576.8 μm, *z* = 24.1 μm of human primary pericytes in MR-NSs; E) 3D acquisition volume of *x* = 576.8 μm, *y* = 576.8 μm, *z* = 31.1 μm of human primary microglia in MR-NSs.

Thanks to the specifically designed geometry of the MR-CSs, the cells in the seeding phase are able to remain inside the MR-CSs, which act as a temporary containment “cage.” The GBM spheroids obtained within MR-CSs were visualized by staining the cytoskeleton of GBM cells, and representative images are depicted in Figure 4A,B (where an array of structures is imaged), and in Figure S5, Supporting Information.

To test whether MR-NSs could support the growth of different cell lines, other cells composing the brain environment were seeded on the scaffolds. In particular, primary human astrocytes, which support blood-brain barrier and neurons maintenance,^[43,44]^ human primary pericytes, typical cells that contact the brain vessels,^[45]^ and human primary brain microglia, which support neurons and exert immune activities,^[46,47]^ were seeded in the MR-NSs. As shown in Figure 4C–E, all these different cell types could grow in the MR-NSs, confirming that this scaffold geometry supports cellular growth; in Figure S6A,B, Supporting Information, we can also qualitatively appreciate as U87-MG cells are able to colonize all the volume of the MR-CSs.

**Figure 5:**
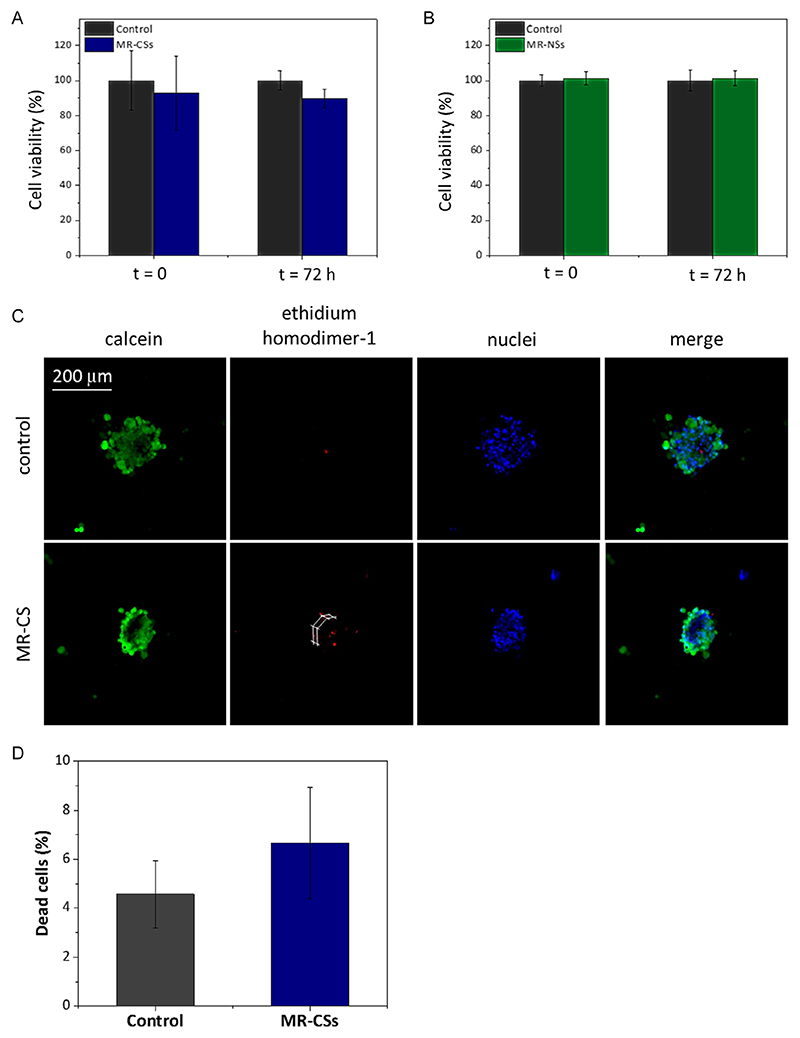
Viability investigation results. A) Cell viability test performed on patient-derived primary GBM cells grown in MR-CSs and in standard conditions at 0 and 72 h (*n* = 5, *p* > 0.05). B) Cell viability test performed on human primary astrocytes grown in MR-NSs and in standard conditions at 0 and 72 h. C) Live and Dead assay acquisitions; to better visualize the presence of the scaffold, white lines were manually drawn. D) Quantification of dead cells (%) with respect to the total number of nuclei (*n* = 5, *p* > 0.05).

Viability tests were carried out to evaluate any difference between traditional cell cultures and cells cultured in the developed magneto-responsive scaffolds. Among all the considered cell lines, for these tests, we focused on primary GBM and astrocytes, being particularly sensitive to the microenvironment conditions. Comparison with GBM traditional spheroids and standard astrocyte cultures has been performed, and results reported in Figure 5A,B show no significant effects of the microstructures on their metabolic activity (*p* > 0.05), as also confirmed by data of the LIVE/DEAD assay reported in Figure 5C,D.

### 3.4 Cell Marker Expression Investigation

**Figure 6:**
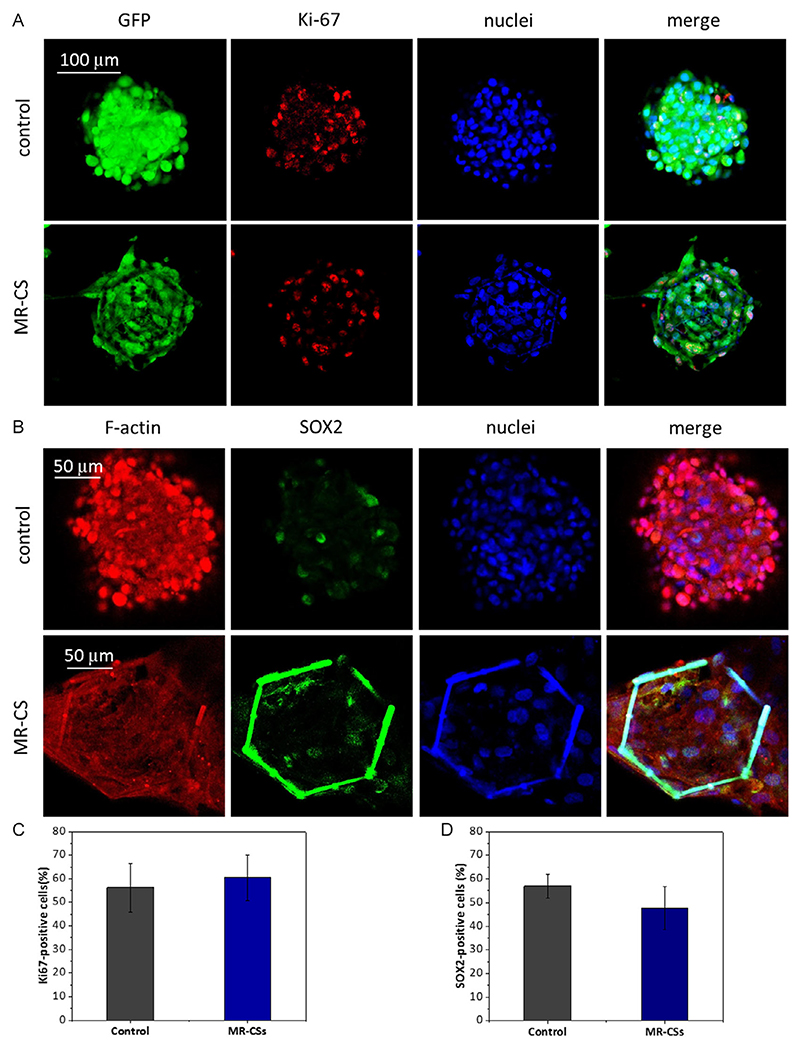
Molecular markers assessment. A) Ki-67 expression (in red) in GFP-expressing U87-MG spheroids obtained in conventional way and within MR-CSs. B) SOX2 expression (green) in primary GBM patient spheroids obtained in conventional way and within MR-CSs. Quantitative analysis of C) Ki-67 (*n* = 5, *p* > 0.05) and D) SOX2 expression (*n* = 4, *p* > 0.05).

To demonstrate the support of the tumor cell growth offered by the MR-CSs, the expression of the Ki-67 was assessed, a proliferation marker routinely used as a clinical prognostic indicator of the proliferative activity of the tumor^[48]^ when characterizing patients’ biopsies. Ki-67 expression is present in actively proliferating cells, and its intense expression is typical of the mitotic phase of the cell cycle; it is also expressed at lower levels in the G1 and S phases, but not in the G0 phase.^[49]^ The confocal acquisitions in Figure 6A show a high Ki-67 expression in the U87-MG spheroids developed inside the MR-CSs (60.4 ± 9% of Ki-67 positive cells), similarly to what was observed in conventional spheroids (56.3% ± 10), without a statistically significant difference (*p* > 0.05), confirming the intense proliferative activity occurring in the scaffolds.

MR-CSs have been shown to be able to support the growth and maintenance of primary GBM patient cells, evaluated by assessing the expression of SOX2, a marker for cancer cell stemness^[40,50]^ (Figure 6B) that suggests maintenance of microenvironment features typical of the physiological tumor niche. SOX2 is one of the fundamental GBM markers, indicative of the self-renewal capacity of cancer stem cells, and its expression in GBM cells is associated with tumor aggressiveness.^[51–54]^ Quantitative analyses show that SOX2 levels in spheroids obtained within MR-CSs are not statistically different from standard 3D cultures (Figure 6C,D, *p* > 0.05).

### 3.5 Migratory Capability

**Figure 7:**
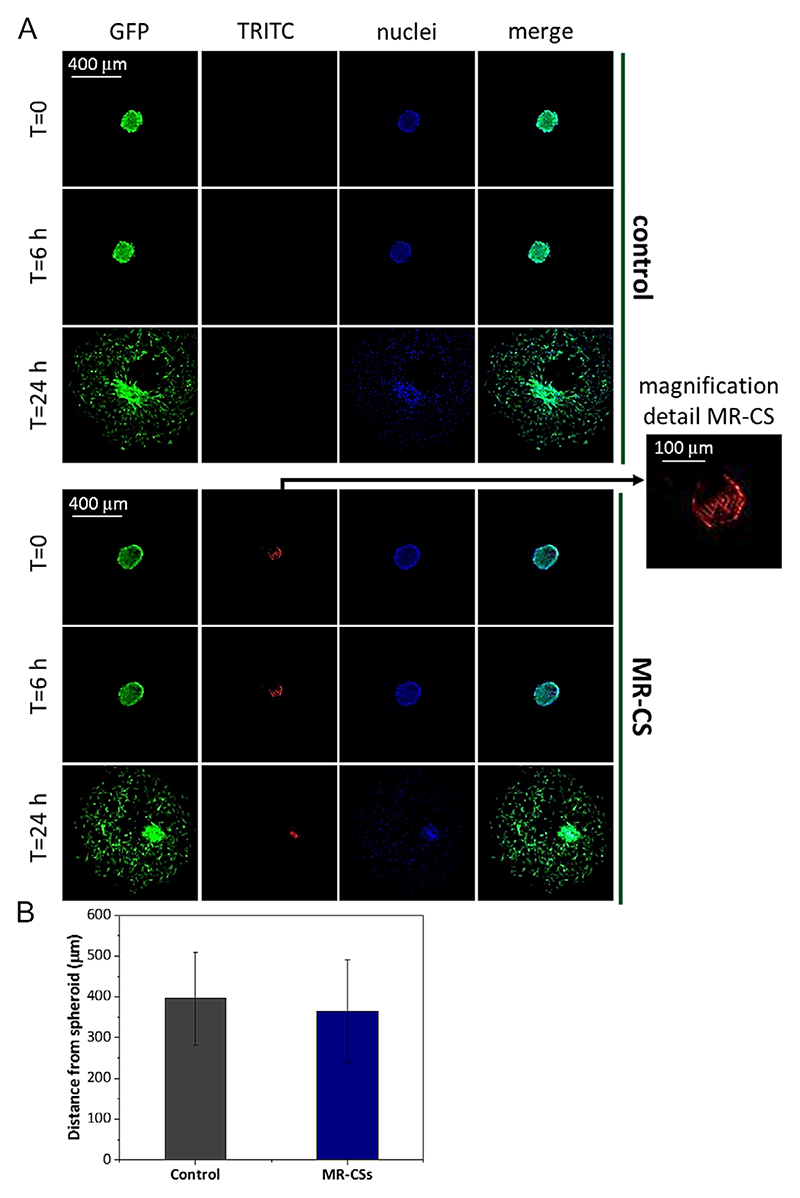
Migration studies results. A) Cell spreading from spheroids of GBM cells: conventional and MR-CSs-based cultures. B) Analysis of the distance (μm) covered by the cells during migration (*n* = 3, *p* > 0.05).

One of the main features of GBM is its ability to invade healthy brain tissues and studying tumor cell invasion following anticancer treatment is a common assay in in vitro research: ensuring that cells within MR-CSs maintain this feature is thus of particular importance. We showed that cells, thanks to the appropriate fenestration in the MR-CS structure, were able to migrate out of the scaffolds (Figure 7). Figure 7A shows representative images supporting the migration and the spreading of cancer cells in the surrounding environment; the observed behavior is analogous to that one occurring in the case of traditional, “free standing” 3D cultures (conventional spheroids: 396 μm ± 113, 181 μm ± 98; MR-CSs: 365 μm ± 125, 337 μm ± 270 -without and with neuron-like cells, respectively-; Figure 7B).

**Figure 8:**
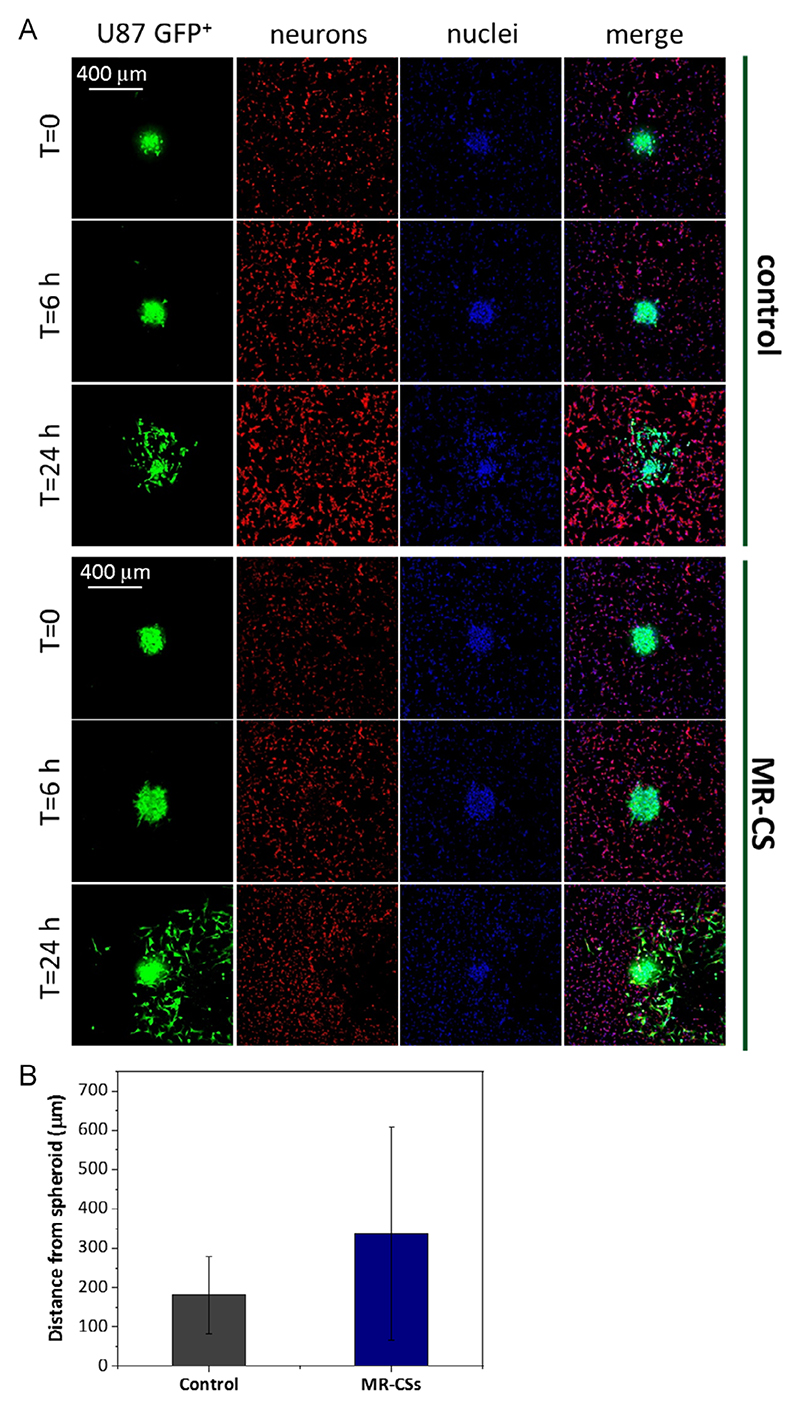
Migration studies in the case of coculture with neuronal-like cells. A) Cell spreading from spheroids of GBM cells: conventional and MR-CSs-based cultures. B) Analysis of the distance (μm) covered by the cells during migration (*n* = 3, *p* > 0.05).

Figure 8 reports results of the same experiment carried out in coculture with neuronal cells, and support data reported in Figure 7.

### 3.6 Magnetic Manipulation

**Figure 9:**
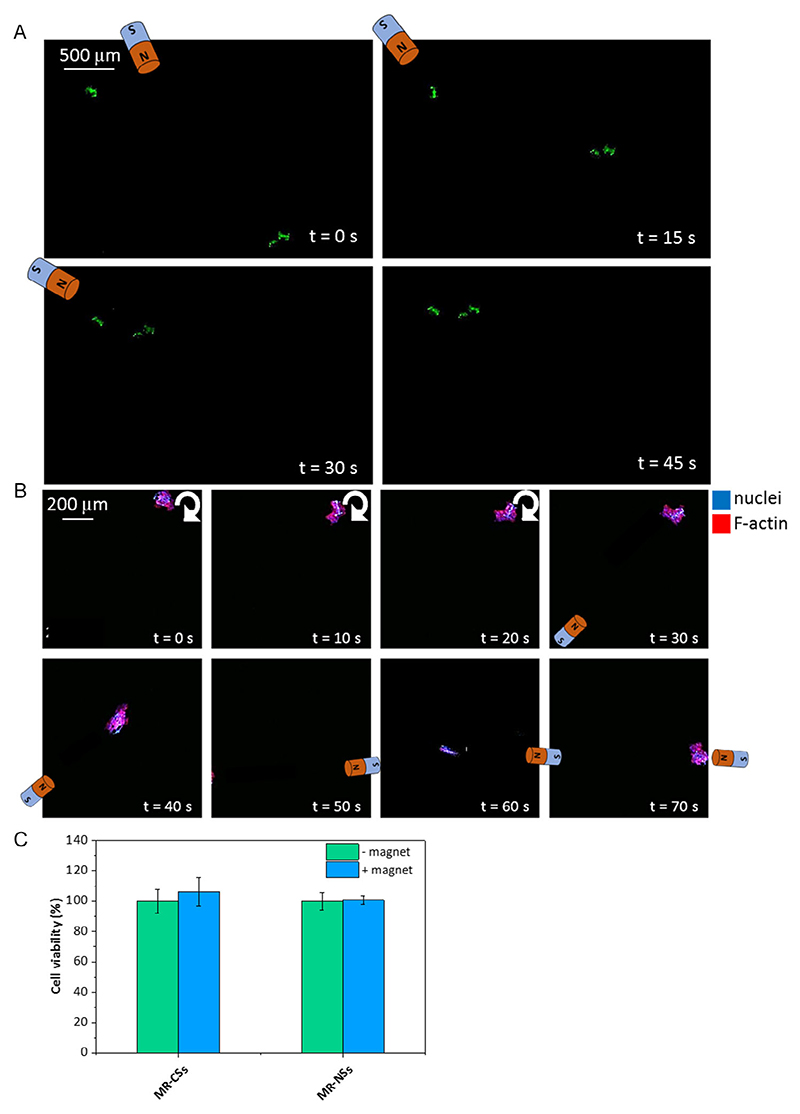
Time-lapse representative images of the movement of magneto-responsive structures. A) MR-CSs brought together by the guidance of an external magnet; B) movement of a U87-MG spheroid in an MR-CS under the action of an external magnet. C) Viability of U87-MG in the MR-CSs and human astrocytes in MR-NSs before and after exposure for 6 h to an external permanent magnet (in both cases *n* = 6, *p* > 0.05).

The proposed scaffolds can be easily manipulated by a remote magnetic field, making them suitable as actual delivery platforms. Figure 9 shows some examples of controlled movements: Figure 9A and Video S1, Supporting Information, report three individual MR-CSs moved by the magnet until forming a tripartite structure; Figure 9B and Video S2, Supporting Information, show the movement of a U87-MG spheroid grown inside the MR-CS, detached from the substrate, and moved by the operator with the help of an external magnet. In Supporting Information, eventually, Figure S7 and Video S3, Supporting Information, show the movement of the “shuttle-shaped” scaffold seeded with GFP-expressing U87-MG cells, demonstrating the versatility of the fabrication protocol.

The cell viability after placement of the permanent magnet exploited for manipulation was also carried out, considering U87-MG MR-CS spheroids and astrocytes growth in MR-NSs subjected to the action of a permanent magnet placed at the bottom of the well for 6 h. The results reported in Figure 9C do not show any significant difference before and after magnet application.

### 3.7 Cocultures Examples

To obtain in vitro predictive systems of the tissue being studied, it is very important to recapitulate its heterogeneity with cocultures of different cells populating the tissue itself.^[55,56]^ Combining cocultures with a 3D approach is a good biomimetic strategy, that makes the in vitro system more similar to the physiological microenvironment.^[57,58]^

**Figure 10:**
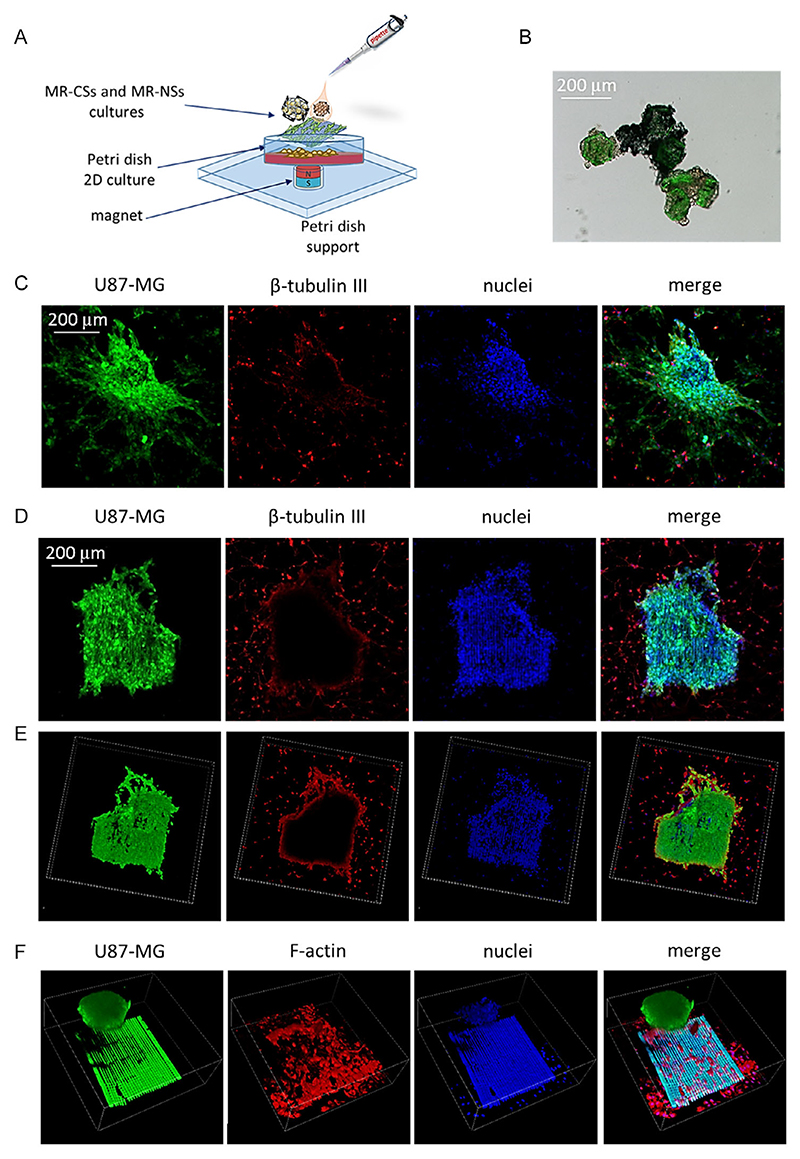
Examples of cocultures obtained with magneto-responsive scaffolds. A) Schematic representation of the assembling procedure. B) Combination, thanks to an external magnet, of U87-MG spheroids obtained within MR-CSs. C) GFP-expressing U87-MG spheroids within MR-CSs placed on a monolayer of human neuron-like cells. D) GFP-expressing U87-MG spheroids within MR-CSs placed on MR-NSs scaffolds bearing GFP-expressing primary human astrocytes; the whole complex is placed on a monolayer of human neuron-like cells. E) 3D rendering with a volume of *x* = 746.1 μm, *y* = 746.1 μm, *z* = 99.4 μm of the system described in D. F) 3D acquisition with a volume of *x* = 578.4 μm, *y* = 578.4 μm, *z* = 202.0 μm of a GFP-expressing U87-MG spheroid within a MR-CS placed on human microglia grown in MR-NSs; the whole complex was placed on a monolayer of human brain endothelial cells.

Figure 10 reports some examples of 3D cocultures obtained by combining MR-CSs with MR-NSs, seeded with several different cell typologies; Figure 10A, in particular, depicts a schema of the seeding procedures.

In the first case, U87-MG spheroids were grown in MR-CSs and then brought together by a magnet placed under the cell culture dish (Figure 10B). This example could be useful in experiments where it is necessary to increase the volume of the GBM in vitro model, for example, to evaluate gas exchange in relation to the tumor mass, to evaluate the diffusion and penetration of drugs, or to follow the evolution of the necrotic mass of GBM that can develop as a result of hypoxia.

The second case, instead, represents a U87-MG spheroid placed on a monolayer of human neuron-like cells, placed in coculture (Figure 10C). It is clearly visible how the GBM cells can migrate from and spread over the surface occupied by the neurons, a typical behavior of cancer cells.^[59]^ As mentioned earlier, the interactions among GBM cells and the healthy brain components play an important role in the development of GBM drug resistance and in the support of tumor growth. It has been demonstrated, for example, that GABAergic neurons in the presence of GBM can release aberrant glutamate secretion in the glioma microenvironment, which promotes tumor progression and contributes to the development of comorbidities, such as cognitive defects, epilepsy, and widespread neurodegeneration.^[60]^ An in-depth understanding of these neuropathological aspects, also thanks to more sophisticated in vitro studies, could lead to improvements in the prognosis of patients with GBM.

The proposed magneto-responsive scaffolds enable 3D growth and direct coculture of diverse cell populations on scaffolds with different geometries, which can be subsequently combined in direct coculture (cell–cell contact), replicating in vitro complex biological systems. Figure 10D–E reports U87-MG cells developed as spheroids, obtained within MR-CS (top), placed on a culture of primary human astrocytes obtained with MR-NSs, which, in turn, are placed on a monolayer of human neurons (bottom). The interaction between GBM and astrocytes is also important in in vitro studies of GBM; in fact, it has already been demonstrated that astrocytes can modulate the chemo-sensitivity of GBM, and support tumor growth by depriving and detoxifying GBM cells from chemotherapeutic drugs.^[61]^

A final example, eventually, reports a 3D coculture of U87-MG cells developed as spheroids within MR-CSs placed on a culture of human microglia in MR-NSs scaffolds, jointly to a monolayer of human endothelial cells (Figure 10F). This could be useful in understanding the interactions between GBM and endothelial cells, and related phenomena such as neoangiogenesis and invasiveness.^[62,63]^

All these studies highlight the importance of multicellular interactions in the glioma microenvironment, and stress the necessity to develop faithful and reliable complex in vitro models that could help in improving drug efficacy predictability and reproducibility, filing the gap between traditional in vitro investigations and preclinical studies.

## 4 Conclusion

In this work, we presented two scaffold models realized with 2-PP technique for advanced 3D cocultures reproducible on a large scale and obtained with a SPIONs-doped photoresist, which confers magneto-responsiveness. It has been shown that the combination of several scaffolds can develop cocultures useful for the study of the human brain environment affected by GBM, with the aim of obtaining more predictive in vitro investigations, that could greatly reduce the in vivo experiments in line with the “3*R*” approach. It has been demonstrated that several cell lines typical of the human brain are able to effectively grow in the scaffolds and that the scaffolds in turn can be detached and moved remotely by using an external magnet, without the need for a “contact” manipulation. The proposed approach, in our opinion, represents an interesting opportunity in the field of microphysiological systems, with the aim of obtaining real-scale, user-friendly, and reliable models of the brain cancer microenvironment.

## Supplementary Material

Supporting information

Video S1

Video S2

Video S3

## Data Availability

The data that support the findings of this study are openly available in Zenodo at https://doi.org/10.5281/zenodo.11085868, reference number [64].
