# Supplementary material for: Remotely Controlled 3D-Engineered Scaffolds for Biomimetic In Vitro Investigations on Brain Cell Cocultures
Source: Adv Intell Syst. Author manuscript; Available in PMC 2024 Sep 19. (PMC7616606; doi:10.1002/aisy.202400261)
Supplement: Supporting information [file EMS197469-supplement-Supporting_information.pdf]

Supporting Information

Remotely controlled 3D-engineered scaffolds for biomimetic *in vitro* investigations on brain cell co-cultures

Daniele De Pasquale[*,1], *Attilio Marino*[1], *Carlotta Pucci*[1], *Omar Tricinci*[1], *Carlo Filippeschi*[2], *Pietro Fiaschi*[3,4], *Edoardo Sinibaldi*[*,2], *Gianni Ciofani*[*,1]

1 Istituto Italiano di Tecnologia, Smart Bio-Interfaces, Viale Rinaldo Piaggio 34, 56025 Pontedera, Italy

2 Istituto Italiano di Tecnologia, Bioinspired Soft Robotics, Via Morego 30, 16163 Genova, Italy

3 IRCCS Ospedale Policlinico San Martino, Department of Neurosurgery, Largo Rossana Benzi 10, 16132 Genova, Italy

4 University of Genova, Department of Neuroscience, Rehabilitation, Ophthalmology, Genetics, Maternal and Child Health (DiNOGMI), Largo Paolo Daneo 3, 16132, Genova, Italy

*Corresponding Authors

E-mail: daniele.depasquale@iit.it; edoardo.sinibaldi@iit.it; gianni.ciofani@iit.it

Cell type	Average diameter (µm)	Standard deviation ± (µm)
U87-MG	9.0	1.7
human astrocytes	11.4	2.7
human pericytes	16.2	3.1
human microglia	9.2	2.8
primary GBM cells	9.1	2.5

Table S1: Average diameter of the cells in suspension, assessed for the design of the scaffolds.

Magneto-responsive micro-scale "space shuttle"

In order to emphasize the versatility of the proposed technique, a "space shuttle" structure has been designed and produced with the same approach described in the main text. Briefly, the micro-scale "space shuttle" presents a length of 122 µm and a width of 77 µm, considering the wings. The height of the fuselage is 20.5 µm and it attached to the glass support thanks to six cubic feet of 2 µm *per* side.

GFP-expressing U87-MG cells (Cellomix) were seeded ($3 \cdot 10^4$ cells in 50 µl complete medium on the "space shuttle" array placed in a 3-cm Petri dish), and after 2 h allowing adhesion, 2 ml of DMEM complete medium was added.

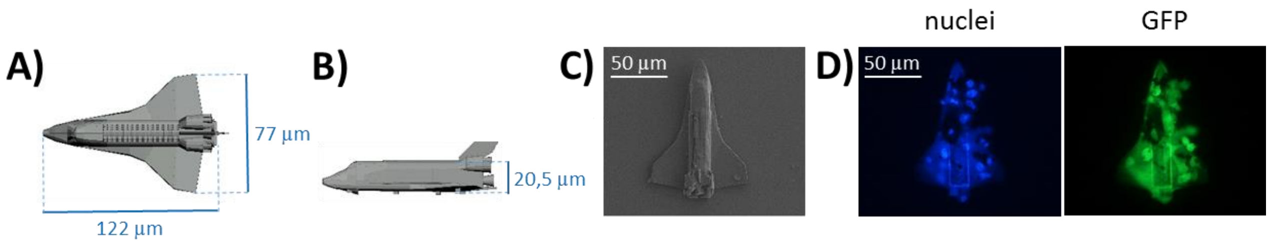

Figure S1. Micro-scale "space shuttle" model. Design of the structure with relative sizes: **A**) top view and **B)** lateral view. **C**) SEM imaging. **D**) Fluorescence microscope imaging of structure bearing GFP-expressing U87-MG cells (nuclei in blue).

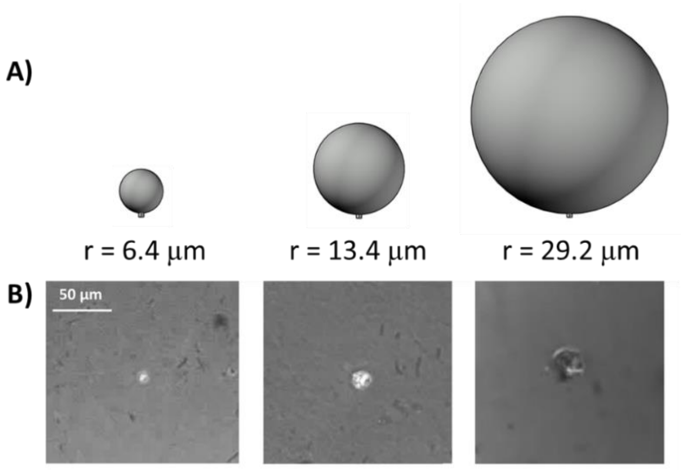

Figure S2. Spheres exploited for the magnetic characterization: **A)** design of spheres with relative sizes; **B)** optical microscope images.

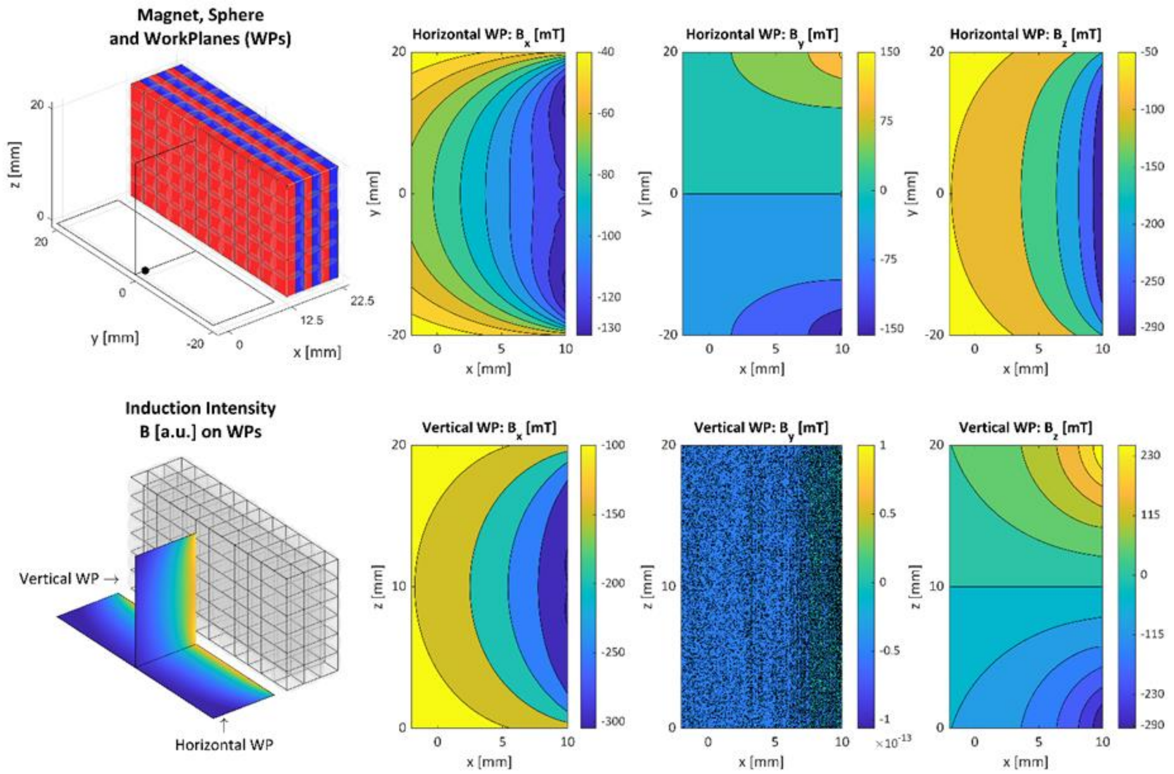

Figure S3. Magnetic induction, as generated by the adopted brick permanent magnet, on illustrative workplanes containing the magneto-responsive sphere. Given a Cartesian (xyz-)frame with origin at the sphere, the 10×40×20 mm^3 brick magnet (aligned with the frame axes) is here discretized by assuming 3×12×6 dipoles (color-hinted in the top-left image, with red nominally associated with magnet north pole). Magnetic induction components B_x, B_y and B_z are shown on the horizontal (xy-) and on the vertical (xz-) workplane (with B_y numerically null on the latter, by symmetry). Induction intensity $B = \left(B_x^2 + B_y^2 + B_z^2\right)^{1/2}$ is also shown on both workplanes, for completeness.

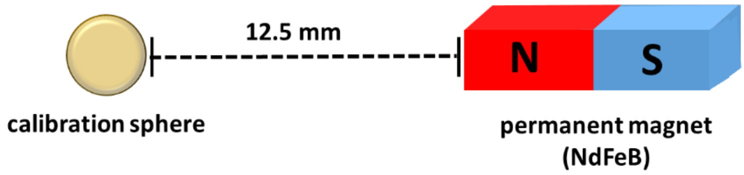

Figure S4. Scheme of the experimental set-up used for video recordings acquired for the characterization of the magnetic properties of the nanocomposite.

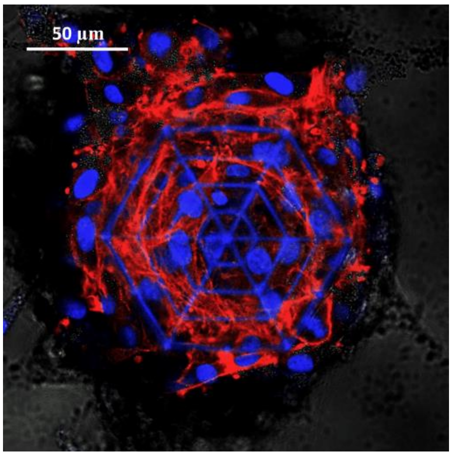

Figure S5. Representative confocal image of a medial plane of a U87-MG cells spheroid obtained within a MR-CS (F-actin in red, nuclei in blue).

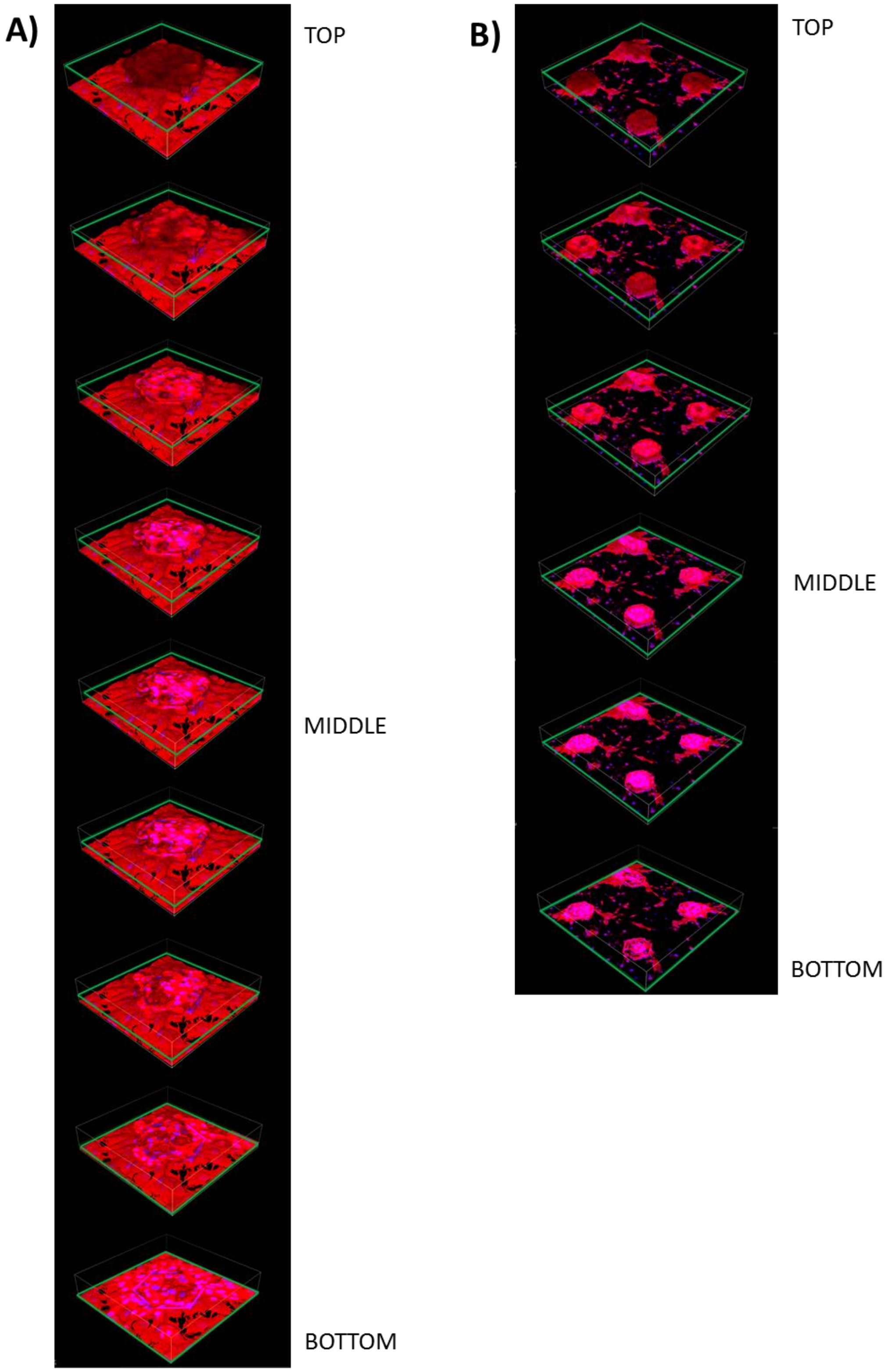

Figure S6. Confocal 3D acquisition with *Z*-stack images (F-actin in red, nuclei in blue). **A)** Representative U87-MG spheroid obtained within a MR-CS (total volume of x = 250.2 µm, y = 250.2 µm, z = 62.1 µm; each Z-step ≈ 7 µm). **B)** Spheroids obtained within a 4-MR-CS array (total volume of x = 636.8 µm, y = 636.8 µm, z = 102.2 µm; each Z-step ≈ 10 µm).

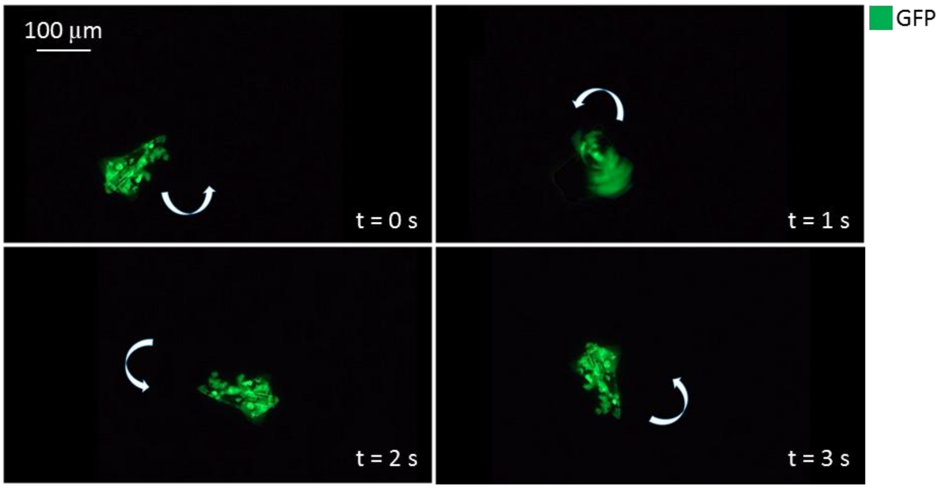

Figure S7. Time-lapse representative images of rotation movements of the micro-scale “space shuttle” under the action of a magnet.

Video S1. Movement of MR-CSs brought together by the guidance of an external magnet.

Video S2. U87-MG spheroid in a MR-CS under the action of an external magnet.

Video S3. Rotation movements of the micro-scale “space shuttle” under the action of a magnet.
